# Risk Factors for Bullying Victimization in Children with Neurofibromatosis Type 1 (NF1)

**DOI:** 10.3390/children8020145

**Published:** 2021-02-15

**Authors:** Peter L. Stavinoha, Cody Solesbee, Susan M. Swearer, Steven Svoboda, Laura J. Klesse, Alice Ann Holland

**Affiliations:** 1 Division of Pediatrics, University of Texas MD Anderson Cancer Center, Houston, TX 77030, USA; 2 College of Education and Human Sciences, University of Nebraska-Lincoln, Lincoln, NE 68508, USA; cody.solesbee@huskers.unl.edu (C.S.); sswearernapolitano1@unl.edu (S.M.S.); svoboda@unl.edu (S.S.); 3 Department of Psychiatry (AAH), Department of Neurology (LJK), University of Texas Southwestern Medical Center and Children’s Medical Center Dallas, Dallas, TX 75235, USA; laura.klesse@utsouthwestern.edu (L.J.K.); Alice.Holland@childrens.com (A.A.H.)

**Keywords:** bullying, neurofibromatosis, social

## Abstract

Neurofibromatosis type 1 (NF1) is an autosomal disorder associated with numerous physical stigmata. Children with NF1 are at known risk for attention-deficit/hyperactivity disorder (ADHD), academic struggles, and significant social difficulties and adverse social outcomes, including bullying victimization. The primary aim of this study was to identify risk factors associated with bullying victimization in children with NF1 to better inform clinicians regarding targets for prevention and clinical intervention. Children and a parent completed questionnaires assessing the bully victim status, and parents completed a measure of ADHD symptoms. Analyses were completed separately for parent-reported victimization of the child and the child’s self-report of victimization. According to the parent report, results suggest ADHD symptoms are a significant risk factor for these children being a target of bullying. Findings for academic disability were not conclusive, nor were findings related to having a parent with NF1. Findings indicate the need for further research into possible risk factors for social victimization in children with NF1. Results provide preliminary evidence that may guide clinicians working with children with NF1 and their parents in identifying higher-risk profiles that may warrant earlier and more intensive intervention to mitigate later risk for bullying victimization.

## 1. Introduction

Neurofibromatosis type 1 (NF1) is an autosomal dominant disorder caused by pathogenic variants in the NF1 tumor suppressor gene [1]. NF1 has an incidence rate of approximately 1 in 2500–3000 live births [2,3]. Clinical manifestations of the disorder include café-au-lait macules, axillary freckling, and neurofibromas and an increased risk of malignancies [4,5]. However, the clinical expression of the disorder varies widely, as some patients demonstrate numerous and prominent physical stigmata whereas others have only mild cutaneous manifestations [6,7]. Attention problems are common, with approximately 30–50% of youth with NF1 diagnosed with comorbid attention-deficit/hyperactivity disorder (ADHD) [5]. Visuospatial difficulties also are common, and there is evidence of language, memory, and executive dysfunction as well [8]. Academic/school problems occur in as many as 75% of children with NF1 [9]. The unique clinical presentation—from physical abnormalities and neurocognitive symptoms to comorbid psychiatric diagnoses—place NF1 youth at an increased risk for adverse social experiences. Indeed, children with NF1 report greater difficulty in making and maintaining social relationships compared to their unaffected siblings [7], and overall, children with NF1 exhibit a high prevalence and severity of social dysfunction [10].

Other adverse social experiences potentially affecting children with NF1 include bullying, which is defined as harmful behavior that is intentional, repeated, and involves a power imbalance between perpetrators and targets [11,12]. A variety of forms and methods are used to perpetrate bullying, including electronic harassment, humiliation, social exclusion, verbal harassment (e.g., taunts and threats), and physical harm [13,14]. In the general population, prevalence of bullying victimization is estimated at about 22% [15,16]. Among students with disabilities, estimates of bullying victimization range from approximately 24 to 34%, with elevated risk of repeated victimization [17]. We previously found that 61.7% of children with NF1 reported being bullied during the previous 12 months [18]. In general, despite the prevalence of bullying among school-age youth, little is known about the risks for bullying victimization in children with NF1, who commonly have a number of characteristics that could make them more likely to be targets of bullying. As applied to children with NF1, the social–ecological diathesis stress model of bullying suggests that concomitant risks such as ADHD, school problems, and having parents with NF1 may increase the stress of bullying victimization [16].

Bullying victimization is associated with health consequences including difficulty with sleep, poor eating habits, depression, and anxiety [19,20,21,22]. The prevalence of reported pain (e.g., stomachaches, headaches, and back pain) is significantly higher in victims of bullying [23]. Indeed, bullying victimization is recognized by the National Academies of Science, Engineering, and Medicine [24] as a serious health concern affecting children and adolescents worldwide.

Prior to Holland et al. [18], bullying victimization had not been specifically examined in youth with NF1. Studies reporting social difficulties in children with NF1 have focused on peer interactions, friendships, and social competence, but not active social rejection defined as bullying [25,26,27]. While recent data indicate high frequency of bullying victimization in children with NF1 [18], the specific risk factors for bullying victimization remain unclear, as social problems in children with NF1 should not be conflated with experiences of bullying victimization. Through secondary and additional analysis of the Holland et al. [18] sample, this study seeks to elucidate risk factors common in NF1 that may result in increased risk for bullying victimization, in order to identify and clarify potential targets for effective prevention and intervention.

### 1.1. Attention-Deficit Hyperactivity Disorder

ADHD symptoms represent a known risk factor for bullying victimization [28,29,30] even above other disability categories [31], in a large part due to aversive and socially inappropriate behaviors that increase the risk for being targeted [28,29,32]. Research linking ADHD and rates of peer rejection has found that children with ADHD experience significantly more victimization than their peers. For instance, Twyman and colleagues [33] found that 29% of youths with ADHD reported experiencing peer victimization, compared to 9% of youths without a psychiatric diagnosis. Youths with ADHD who have a history of being bullied displayed significantly more internalizing and externalizing problems in self- and parent-reports [30]. Hyperactive/impulsive behaviors of ADHD tend to be associated with bullying victimization—including the persistence of bullying victimization over time—more than inattention symptoms are [34].

Symptoms of ADHD are common in children with NF1, with 30–50% of children with NF1 exhibiting attention deficits, compared to 3–7% of their peers in the general population [35,36]. It follows that children with NF1 and ADHD symptoms are at greater risk for poorer social outcomes, which may include bullying victimization, compared to children with both NF1 without ADHD [25]. Noll and colleagues [27] included ADHD in their definition of neurological severity in NF1 and found a significant association with social difficulties. However, this study did not examine active social rejection or bullying in NF1, and neurological severity was inclusive of many factors beyond ADHD—such as learning disabilities, seizures, and brain tumors—thereby confounding the specific relationship between ADHD and social difficulties in their sample. Thus, the relationship between NF1, ADHD symptoms, and bullying victimization has not yet been examined.

### 1.2. Academic Disability

NF1 negatively affects specific domains of cognitive functioning, such that 30–75% of youths have learning difficulties and poor school performance [36,37,38,39]. In the academic setting, students with disabilities are significantly overrepresented within the bullying dynamic [40,41,42,43], and disability status has been shown as a predictor for increased victimization [16,42,44,45]. Students with learning difficulties are 3–3.5 times more likely to experience bullying victimization and rejection [46,47,48] and are viewed as less socially competent than peers without learning difficulties [49]. The extant literature suggests that students with disabilities experience elevated rates of victimization longitudinally across their academic years [50,51,52]. While bullying victimization as a function of academic competence in NF1 has not been investigated, there is some evidence of greater social dysfunction in children with NF1 being associated with more significant learning difficulties [27], though this study included learning disabilities as part of a broader variable reflective of neurological symptom burden and did not include active social rejection or bullying victimization as outcomes.

### 1.3. Parent with NF1

Studies of child development have demonstrated that parents contribute to their children’s peer relationships through both direct (e.g., skill coaching) and indirect (e.g., parental attitudes) pathways [53]. Thus, parental influence on children’s peer relations may also be impacted by parental social competence. As adults with NF1 are at increased risk for deficits in social skills and competence [54], having a parent with NF1—which occurs in approximately 50% of cases of NF1 [55]—might be an additional risk factor for social deficits. Indeed, parents with NF1 often report smaller social networks and feelings of loneliness [56,57]. Adults with NF1 also consistently report low self-confidence and elevated worry about the progression of the disorder [56,58]. Further, rates of psychiatric illnesses such as depressive conditions are more frequent among adults with NF1 [57,59]. In these ways, having a parent with NF1 may represent a risk factor for poor development of social skills among youths with NF1, thereby increasing their risk for bullying victimization.

### 1.4. Factors Excluded from Analysis

Several factors were excluded from analysis due to existing empirical evidence suggesting no significant effect, which the initial study with this sample to establish the rate of bullying victimization in NF1 confirmed. For instance, while it has been suggested that older children may be identified as having more social difficulties relative to preschoolers [10], age was not associated with higher rates of victimization in this sample [18]. Findings of social difficulties according to gender in children with NF1 have been inconsistent, with a recent systematic review concluding only a slightly higher risk for social difficulties in males with NF1 [10]. No gender differences were found in the present sample [18], so gender was not included as a risk variable in the present analysis. Finally, in the present sample there were no significant differences in the frequency of bullying victimization based on visible stigmata of NF1 [18], which is consistent with previous literature finding no significant relationship between social difficulty and visible stigmata of NF1 [10,26,27]. Thus, the present study was designed to extend previous work by investigating novel variables that might be more significant risk factors for bullying victimization in children with NF1.

### 1.5. Purpose of the Present Study

Given the lack of clarity regarding clinically defined targets for prevention and clinical intervention in children with NF1 known to be at high risk for bullying victimization, the primary aim of the present study was to identify relevant clinical risk factors for bullying in children with NF1. Based on the extant literature, the present study included the following known risk factors for bullying victimization and/or social dysfunction: impulsive/hyperactive ADHD symptoms, academic disability (identified by the presence of either individualized education program (IEP) or Section 504 Accommodation Plan), history of grade retention, and having a parent with NF1. Since age, gender, and presence of visible stigmata have not been identified in previous research as significant risk factors associated with social deficits and rejection in children with NF1, these were excluded from the present analysis. We hypothesized that impulsive/hyperactive ADHD symptoms would significantly increase the risk for bullying victimization in children with NF1 more so than other factors, given the known relationship between ADHD and bullying victimization in populations without NF1. As described above, academic disability is also a known risk factor for bullying victimization in children without NF1, so we hypothesized that academic disability would add to the risk for bullying victimization in NF1. Examination of grade retention and having a parent with NF1 as specific risk factors for bullying in children with NF1 were considered more exploratory given a relevant theoretical context yet paucity of literature in these areas.

## 2. Materials and Methods

### 2.1. Participants

Participants were recruited through the comprehensive neurofibromatosis clinic in the oncology department of a major pediatric medical center with a catchment area encompassing multiple states. The study was approved by the Institutional Review Board (IRB) affiliated with that medical center. Study procedures were conducted in compliance with IRB regulations and the ethical standards of the American Psychological Association (APA).

Inclusion criteria were the clinical diagnosis of NF1, proficiency in English, age 8:0 to 18:11, and enrollment in the third grade or above. Exclusion criteria included active chemotherapy treatment, deficits that would prohibit measure completion (e.g., substantial visual impairment; gross cognitive deficits as identified by parent report; and/or research team observation that the child was unable to complete study measures), and physical disfigurement unrelated to NF1.

Qualified patients, identified by the medical chart review, were recruited upon arrival for a routine follow-up visit at the medical center’s Neurofibromatosis Clinic. A total of 81 participants (parent/guardian and child dyads) were consented, enrolled in the study, and completed all study procedures. After screening for eligibility and gathering informed consent and child assent, participants were administered their respective questionnaires. Research staff monitored questionnaire completion and every effort was made to have parent and child complete measures in separate clinic rooms.

### 2.2. Measures

Patient history form: Each parent/guardian completed this 34-item questionnaire, which was developed by the researchers and queried parents/guardians about their child’s relevant history. For the present study, data regarding ethnicity, history of school retention, and current school services (i.e., Section 504 plan or Individualized Education Program) were collected via this questionnaire. Any report of a child receiving formal special services at school was coded as “academic disability.” Grade retention was coded as a separate variable.

The bully survey—parent version (BYS-P) [60]: The current study used parent-report data from Part A, Item 1a of the BYS-P. Responses to a question regarding whether their child has been bullied in the past year were recorded as a dichotomous variable (yes or no), thereby establishing bullying victimization status for the sample. The BYS-P was developed in the United States using principal components factor analysis that yielded a two-factor solution with items loading onto physical or verbal bullying, with no cross-loadings. The BYS-P has demonstrated adequate internal consistency reliability (coefficient alpha = 0.71) and satisfactory test–retest reliability [60,61].

The bully survey—student version (BYS-S) [60]. On this four-part questionnaire, students respond to questions about their experiences with, perceptions of, and attitudes toward bullying. To determine agreement between parent- and self-report of bullying victimization, Part A, Item 1a of the BYS-S was used. Responses to a question whether the child has been bullied in the past year were recorded as a dichotomous variable (yes or no) and compared to the parent report.

Swanson, Nolan, and Pelham–IV Questionnaire (SNAP-IV): The 18-item version of the SNAP-IV [62] is a questionnaire that allows parents or teachers to rate children on a comprehensive selection of symptoms of attention-deficit/hyperactivity disorder (ADHD). Parent ratings on the SNAP-IV 18-item version have been shown to have useful accuracy in differentiating children who meet the diagnostic criteria for AD/HD from those who do not [63]. The SNAP-IV demonstrated optimal internal consistency reliability (Cronbach’s alpha = 0.89–0.94) and satisfactory predictive reliability [61,63]. For the current study, a parent/legal guardian was asked to select a response that best described their child for each item on a 4-point Likert scale (0 = “not at all” to 3 = “very much”). The SNAP-IV yields three composite scores reported as continuous variables: inattention, hyperactivity-impulsivity, and combined. The hyperactivity-impulsivity score was utilized for the purpose of the present investigation as it represents a spectrum of potentially aversive and socially inappropriate behaviors known to increase the risk for bullying victimization [29,32,64].

### 2.3. Analysis Procedures

SPSS Version 26.0.0 was used for all analyses. Prior to conducting study analyses, data were screened for multivariate normal distribution, linearity, and outliers; no issues were identified. Analysis of frequencies determined that one case had a missing value for a parent with NF1; therefore, data from 80 participants were used for the analysis.

The SNAP hyperactivity/impulsivity factor was used to represent externalizing ADHD symptomology and was included as a continuous variable. Having a parent with NF1, history of grade retention, and academic disability were coded as dichotomous variables.

## 3. Results

Findings of descriptive statistical analyses regarding sample demographics (i.e., age, gender, and ethnicity), medical factors (i.e., height, weight, and disease severity), and rate and frequency of both parent-reported and self-reported bullying victimization were previously published in Holland et al. [18]. Selected descriptive statistics relevant to the present investigation are summarized in Table 1 for reader convenience.

Self- and parent/guardian reports of bullying were in concordance in 80.3% of participants. Two logistic regression analyses were performed to investigate the relative contributions of four theoretical risk factors—ADHD symptomology, having a parent with NF1, history of grade retention, and academic disability—on parent/guardian-reported history of their child’s bullying victimization (i.e., “victim” or “non-victim” status). Parent/guardian reports of bullying were used to ensure consistency and accuracy across variables given parents’ respective knowledge of their child’s ADHD symptomology, having a parent with NF1, history of grade retention, and academic disability. Table 2 and Table 3 summarize descriptive statistics on variables of interest based on parent-reported history of bullying victimization and separately when subjects are grouped by child-reported history of victimization.

The logistic regression model for parent-reported history of their child’s bullying victimization fit the data, $\chi^{2}$ (4, 80) = 10.74, *p* = 0.030, Nagelkerke $R^{2}$ = 0.17. The model correctly classified 61.8% of bully–victim non-cases and 78.3% of bully–victim cases. The overall classification success rate was 71.3%.

The logistic regression model for child-reported history of bullying victimization did not fit the data well, $\chi^{2}$ (4, 80) = 4.97, *p* = 0.288, Nagelkerke $R^{2}$ = 0.08. The model correctly classified 19.4% of bully–victim non-cases and 85.7% of bully–victim cases. The overall classification success rate was 60.0%.

Table 4 and Table 5 show the regression coefficients and their standard errors, Wald statistics, and odds ratios for each of the predictors for both parent-report and child-report.

Holding everything else constant, the parent-report SNAP hyperactivity/impulsivity was the only predictor distinguishing the bully–victim status (Wald $\chi^{2}$ = 5.37, *p* = 0.021). In other words, the likelihood of being classified as a bully–victim case is multiplied by 2.31 for each one-unit increase in the SNAP hyperactivity/impulsivity rating. Odds ratios suggest potential increased vulnerability associated with grade retention and having a parent with NF1, though statistical significance was not reached. For the child-report, the odds ratios suggested increased vulnerability by having a parent with NF1, though statistical significance was not reached for the overall model.

As an exploratory analysis, the parent–child dyads that were concordant in their reporting of bullying victimization (*n* = 65) were analyzed separately. This analysis is considered exploratory as there is not literature to confirm that parent–child concordance equates to greater accuracy and validity of bully victimization reporting, and therefore such analysis may be overly restrictive in terms of omitting actual cases of bullying victimization. Of the discordant dyads, ten children reported bully victimization whereas their parent did not, and six parents reported bullying victimization whose child did not. Table 6 summarizes demographic information for both concordant and discordant reporting dyads.

As summarized in Table 7, the logistic regression model for concordant dyads reporting history of bullying victimization did not fit the data well, $\chi^{2}$ (4, 65) = 9.106, *p* = 0.058, and Nagelkerke $R^{2}$ = 0.178. The model correctly classified 40.0% of bully–victim non-cases and 87.5% of bully–victim cases. The overall classification success rate was 69.2%.

## 4. Discussion

Overall, the risk model results based on the parent report indicate that behavioral manifestations consistent with symptoms of ADHD are the most significant factor in predicting bullying victimization within this sample of youth with NF1. Higher ratings of ADHD symptoms more than doubled the odds of bullying victimization based on the parent report, and for the child report of bully victimization, odds of reported victimization increased by 1.60 times based on ADHD symptom ratings. This is consistent with previous studies demonstrating that ADHD symptoms are associated with peer victimization [28,30]. The present findings are similar to Barton and North’s [25] findings that children with NF1 and comorbid ADHD are at a greater risk for poorer social outcomes than children with NF1 without ADHD. Our findings extend this to include bullying victimization among adverse social outcomes experienced by children with NF1 and ADHD symptoms.

Having a parent with NF1 was not a statistically significant predictor of bullying victimization in any of the groupings based on the child and parent report. To the best of our knowledge, this is the first study that specifically investigated the impact having a parent with NF1 on bullying victimization in children with NF1. Thus, little is known specifically about this as a possible risk factor. While results were not significant in our sample, a rationale for increased risk is supported by aspects of the social–ecological diathesis stress model of bullying [16]. Specifically, family characteristics, such as having a parent with NF1 and associated psychosocial challenges, represent a microsystem factor that influences bullying involvement [65]. Further, child development literature supports the influence of parenting on important competencies such as social skills [53]. Since previous studies have found that adults with NF1 demonstrate greater social skill deficits [54], it may be that increased risk of bullying victimization is partly a byproduct of lower quality social modeling and possibly an innate, heretofore undefined social deficit common in individuals with NF1. Due to power limitations as discussed further below, the present findings should not be considered definitive but may help guide future research examining risk factors for social deficits and victimization in the NF1 population.

The academic setting was examined as an additional microsystem, which may be related to bullying victimization. Within this domain, academic disability (i.e., presence of an IEP or Section 504 plan) and grade retention were explored as separate constructs. Results did not reach statistical significance for the parent or child report of bullying victimization. As with having a parent with NF1, there remains a rationale to further investigate academic disability as a risk factor given limitations associated with a single site study of a rare disease. Previous literature has found that the disability status is a predictor of increased victimization [44,45,46]. For instance, youth with specific learning disabilities (SLDs) are 3.50 times more likely to experience bullying victimization compared to their non-SLD peers [46,47]. Given that an estimated 30–75% of youth with NF1 have learning disabilities and impaired academic performance, it remains plausible that academic disability-related bullying is a common adverse experience for this population. Similarly, grade retention may be a process that “others” youth with NF1, whereby someone becomes a target for bullying because they are perceived as different compared to their peers [66], though the fact that odds for bully victimization based on grade retention were not increased for the child report of bully victimization introduces caution in premature interpretation of this finding.

Cognitive and academic deficits that lead to special needs designation for children with NF1 may reflect information processing difficulties that contribute to their social difficulties, including increased risk for victimization. While cognitive variables were not part of the present study, there is evidence that children with information processing difficulties such as those common in children with NF1, including nonverbal processing difficulties and executive function deficits, may demonstrate increased vulnerability [67,68]. Whether risk increases as a function of observable socially aversive behaviors or processing deficits that decrease social conflict resolution skills remains to be determined.

In terms of clinical implications, results from this investigation provide preliminary evidence pointing to ADHD symptoms as a primary intervention target for those working with youth with NF1, not only to manage the emotional, behavioral, and academic impacts of these difficulties [69], but also to mitigate social risk for bullying and peer rejection. Given the high prevalence of ADHD symptoms in children with NF1, proactive and immersive intervention to decrease social vulnerability may be warranted, similar to that supported in the ADHD literature [70]. Even though our findings did not reach statistical significance for our sample, there is ample empirical evidence outside of the NF1 literature to suggest that clinicians and educators should take particular care to screen/monitor for any bullying victimization in children who have been designated as having an academic disability or have been retained. Indeed, these findings may influence retention decisions for students with NF1 given potential for added social risk.

Several limitations and directions for future research are recognized. First, measurement of bully victimization status relied upon parent and child response to a single item on a validated scale. While not preferred when measuring complex constructs, single item measures have utility when measuring homogenous concepts and experiences [71]. Since we were interested in the binary option of whether or not a child had been victimized, and not other dimensions of the experience, the single-item option was chosen.

A second limitation of measurement is the lack of other informants (e.g., peers, teachers), which may have offered independent validation of a child’s bully victimization status. Reliance on the parent or child report leaves open possibilities of bias or distortion in reporting of an experiential variable such as bully victimization. Indeed, a recent review calls into some to question children’s capacity for self-reporting, with those with neurodevelopmental disorders, including ADHD and learning disorders demonstrating lower validity of self-report [72]. Further, parent–child agreement in reporting bully victimization is low, and lower still in the context of hyperactive/impulsive symptoms [73], further supporting the need for additional sources of information regarding a child’s bully status. There may be parent–child relationship and interaction factors that promote or inhibit child reporting of bullying victimization, and parent characteristics that lead to increased or decreased reporting. Unfortunately, there is no gold standard for measuring bullying victimization [74], and imprecision of measurement of bully victimization may lead to erroneous results in the analysis of risk factors. Validity issues with reporting of bullying victimization are a challenge that has yet to be conquered in the bullying literature. The clearer the picture of legitimate risk factors for bully victimization in children with NF1, the more likely proactive and early intervention can be mobilized for those youth with higher likelihood of bully victimization.

The lack of a comparison group without NF1 warrants caution in making attributions of risk directly back to NF1 without further validation. To better understand the risk profile for bullying victimization among youth with NF1, and what risks may be different in NF1 compared to other populations, a matched-sample control group should be considered in future research. Additionally, more work is necessary to identify the nature of the heightened risk for bullying victimization in children with NF1 who also have a parent with NF1. Examining offspring with and without NF1 who have a parent with NF1 might help separate innate social difficulties associated with NF1 from family social environmental influences, which would have significant implications for intervention.

Design issues constitute another limitation. The sample was recruited through a multidisciplinary NF clinic at a single medical center and may not be representative of the full spectrum of children with NF1. Further, while the sample size for this study is relatively large compared to many studies of NF1, the size of the sample impacted our power to model risk factors and limited the number of variables to consider individually and in combination. The complexities of contributing factors to bully victimization in children with NF1—ranging from behavioral characteristics to social environmental variables to innate cognitive patterns –warrant a multisite approach to optimize sampling for a clarity of results and more definitive conclusions. While our study does not provide firm answers, it clearly justifies further research into the underpinnings of bully victimization in children with NF1.

Better understanding the impact bullying has on children with NF1 will be important for optimal and timely clinical intervention. While efforts to mitigate risk of social rejection should be enhanced, risk will certainly remain. Understanding the emotional and social burden of bullying victimization in children with NF1 may help highlight targeted interventions for coping, skill development, and healthy psychosocial adjustment.

## Figures and Tables

**Table 1:** Demographic characteristics of sample.

Characteristic	Min.	Max.	*M*	*SD*
Age (years)	8	18.11	11.94	2.79
Characteristic			*n*	%
Ethnicity				
Caucasian			49	60.5
Hispanic			17	21.0
African American			11	13.6
Asian			4	4.9
Gender				
Male			40	49.4
Female			41	50.6

**Table 2:** Risk factor frequencies by the parent-reported group.

Group	Parent with NF	Academic Disability	Retention History	SNAP Rating
Overall Sample (*n* = 80)	33 (41%)	48 (60%)	17 (21%)	1.04 (0.76)
Victims (*n* = 46)	22 (48%)	43 (93%)	12 (26%)	1.22 (0.76)
Non-Victims (*n* = 34)	11 (31%)	25 (71%)	5 (14%)	0.80 (0.70)

**Table 3:** Risk factor frequencies by child-reported group.

Group	Parent with NF	Academic Disability	Retention History	SNAP Rating
Overall Sample (*n* = 80)	33 (41%)	62 (77%)	17 (21%)	1.04 (0.76)
Victims (*n* = 49)	23 (46%)	31 (63%)	11 (22%)	1.18 (0.78)
Non-Victims (*n* = 31)	10 (32%)	17 (55%)	6 (19%)	0.81 (0.78)

Note: Percentages reported for victims and non-victims reflect the percentage within each subgroup represented by the raw number. SNAP hyperactivity-impulsivity rating data are presented as mean scores, with standard deviation in parentheses.

**Table 4:** Logistic regression analysis of parent report of their child’s bully victimization.

Predictor	*β*	*SE*	Wald *χ^2^*	Odds Ratio	*p*	95%CI
Constant	−1.08	0.52	4.31	0.34	0.038	
SNAP Rating	0.84	0.36	5.37	2.31	0.021	[1.14, 4.68]
Parent with NF1	0.68	0.50	1.87	1.98	0.172	[0.74, 5.28]
Grade Retention	0.71	0.62	1.31	2.03	0.252	[0.60, 6.84]
Academic Disability	0.24	0.50	0.23	1.27	0.634	[0.48, 3.38]

**Table 5:** Logistic regression analysis of child report of bully victimization.

Predictor	*β*	*SE*	Wald *χ^2^*	Odds Ratio	*p*	95%CI
Constant	−0.65	0.59	1.20	0.53	0.273	
SNAP Rating	0.47	0.41	1.29	1.60	0.255	[0.71, 3.56]
Parent with NF1	0.71	0.46	2.04	2.03	0.153	[0.77, 5.35]
Grade Retention	−0.06	0.61	0.01	0.94	0.919	[0.29, 3.09]
Academic Disability	0.39	0.55	0.51	1.48	0.477	[0.50, 4.35]

**Table 6:** Demographic characteristics of concordant and discordant reporting pairs.

Characteristic	Concordant (*n* = 65)	Discordant (*n* = 16)
Age (SD)	12.05 (2.77)	11.40 (3.02)
Gender (% female)	50.7	46.67
Parent with NF1 (%)	43.07	33.33
Ethnicity (%)		
Caucasian	60.00	60.00
Hispanic	24.61	6.67
African American	10.77	26.67
Asian	4.62	6.67

**Table 7:** Logistic regression analysis of concordant parent/child reports of bully victimization.

Predictor	*β*	*SE*	Wald *χ^2^*	Odds Ratio	*p*	95%CI
Constant	−1.32	0.73	3.30	0.27	0.07	
SNAP Rating	0.65	0.46	1.97	1.92	0.16	[0.77, 4.76]
Parent with NF1	0.97	0.58	2.80	2.64	0.10	[0.85, 8.24]
Grade Retention	0.24	0.70	0.12	1.28	0.73	[0.32, 5.05]
Academic Disability	0.78	0.61	1.62	2.18	0.20	[0.66, 7.28]

## Data Availability

The data presented in this study are available on request from the corresponding author. The data are not publicly available.
